# A New Treatment for Cystitis and Irritable Bladder of Females

**Published:** 1873-04

**Authors:** M. Yarnall

**Affiliations:** St. Louis


### A NEW TREATMENT FOR CYSTITIS AND IRRITABLE BLADDER OF FEMALES.

BY M. YARNALL, M.D., ST. LOUIS.

Among the patients presented for treatment at the Gynæcological Clinic of Dr. T. L. Papin, at the Institute for the Treatment of Diseases of Women, at the corner of Twenty-third and Morgan streets, in this city, since June 1, 1871, there were twelve suffering from tenesmus of the bladder, which, with eight cases occurring in Dr. Papin's private practice, makes twenty cases of this troublesome and obstinate class of affections treated by him within the time specified. Of the twelve treated at the clinic, two were brought there by gentlemen in whose practice they occurred, and one was admitted to the Institute, for a short time, for treatment, during the temporary absence of her medical attendant from the city.

It is not my purpose to enter into a description of the various methods of treatment that have been resorted to for the relief of cystitis, or irritability of the bladder of females; but I desire to call the attention of the profession to a treatment practiced by Dr. Papin, in the above referred-to cases, that has afforded such excellent results as to justify the belief that it will supersede all other methods yet devised.

This treatment, which is original with Dr. Papin, consists in dilating the urethra with a long pair of dressing forceps to such extent as to produce a temporary incontinence of urine by rupturing a few of the fibres of the sphincter of the bladder, and repeating the operation, when necessary, at intervals of a week or more until the patient is entirely relieved. In nearly every instance the relief afforded is almost immediate, but in the course of a few days the irritability of the bladder usually returns, when the operation has to be repeated, and, if necessary, again repeated, until a cure is accomplished. No fear need be entertained, in performing this operation, of permanently impairing the function of the sphincter muscles; its restoration is as certain and speedy as when the sphincter ani is ruptured for the cure of fissure of the anus. In several of the more severe cases of those operated on, both the mucous membrane and the muscular fibres were lacerated. The incontinence usually continues for two or three days only.

The operation being at first very painful, it will usually be found necessary, in performing it the first time, to place the patient under the influence of an anæsthetic, but its subsequent performance being much less severe, as a rule, the anæsthetic will be unnecessary unless the patient be of a very nervous temperament. In one case operated on, the irritability of both the bladder and urethra was so great that merely touching the meatus urinarius produced a severe nervous paroxysm. This patient was operated on but once, and was entirely relieved, although she had been a sufferer for ten years, and during that period been subjected to all the usual methods of treatment without benefit.

Of the twenty cases thus operated upon, one had cystitis, with antiversion and flexion of the womb; two had cystitis, with slight antiversion of the womb; one had cystitis, with slight prolapsus, and retroversion of the womb; one had cystitis (a young girl), with no apparent uterine complication; five had irritable bladder, with antiversion of the womb; two had irritable bladder, with antiversion and flexion of the womb; six had irritable bladder, with retroversion of various degrees; and two had irritable bladder, with no displacement of the womb.

In one case the dilatation has been repeated four times, and the patient, though much benefited, is still under treatment. Of five cases, each of whom were operated upon three times, four are cured and one is better, but it is difficult to determine to what extent she was benefited by the operation on account of the excessive use of anodynes for the relief of her nervous symptoms. Of five, each operated upon twice, three are cured, and two still under treatment are benefited. Of seven, upon whom one operation only has been performed, three are cured, and three still under treatment are benefited.

In thirteen of the cases there were uterine lesions of various kinds requiring treatment, and no doubt the use of suitable pessaries, where the vesical trouble was superinduced or aggravated by the uterine misplacement, has added much to the permanent relief afforded. Ten of these cases, although relieved of the vesical affection, are still under treatment for the uterine trouble.

As to the *simplicity* of the operation, as compared with that of the making of a vesico-vaginal fistula, which has been resorted to for securing the same object, viz., incontinence of urine, and thereby affording rest to the inflamed organ, there can be but one opinion. As to its *efficacy*, the records of the clinic, and the result in Dr. Papin's private practice, afford conclusive evidence.

Of the twenty cases thus far treated, eleven have been entirely relieved, and eight, still under treatment, have been benefited, with a prospect of permanent relief. In one only, a very hysterical individual above referred to as addicted to the excessive use of narcotics, and with whom the trouble was probably in the over-wrought mental condition of the patient, rather than the vesical irritation, has it failed; and even here the patient was unquestionably benefited. Certainly, if futher experience confirms the most satisfactory results thus far obtained, this operation may be regarded as almost a specific in this troublesome, and often most intractable, form of disease. Of course, its performance does not preclude the use of such adjuvants as may be deemed necessary.—*Medical Archives.*

